# Acemetacin cocrystal structures by powder X-ray diffraction

**DOI:** 10.1107/S2052252517002305

**Published:** 2017-03-08

**Authors:** Geetha Bolla, Vladimir Chernyshev, Ashwini Nangia

**Affiliations:** a School of Chemistry, University of Hyderabad, Prof. C. R. Rao Road, Central University PO, Hyderabad 500 046, India; b Department of Chemistry, M. V. Lomonosov Moscow State University, 1–3 Leninskie Gory, Moscow 119991, Russian Federation; c A. N. Frumkin Institute of Physical Chemistry and Electrochemistry RAS, 31 Leninsky Prospect, Moscow 119 071, Russian Federation; d CSIR-National Chemical Laboratory, Dr Homi Bhabha Road, Pune 411 008, India

**Keywords:** crystal engineering, co-crystals, molecular crystals

## Abstract

Cocrystals of acemetacin drug (ACM) with nicotinamide (NAM), *p*-aminobenzoic acid (PABA), valerolactam (VLM) and 2-pyridone (2HP) were prepared by melt crystallization and their X-ray crystal structures determined by high-resolution powder X-ray diffraction. The powerful technique of structure determination from powder data (SDPD) provided details of molecular packing and hydrogen bonding in pharmaceutical cocrystals of acemetacin. ACM–NAM occurs in anhydrate and hydrate forms, whereas the other structures crystallized in a single crystalline form. The carboxylic acid group of ACM forms theacid–amide dimer three-point synthon *R*_3_^2^(9)*R*_2_^2^(8)*R*_3_^2^(9) with three different *syn* amides (VLM, 2HP and caprolactam). The conformations of the ACM molecule observed in the crystal structures differ mainly in the mutual orientation of chlorobenzene fragment and the neighboring methyl group, being *anti* (type I) or *syn* (type II). ACM hydrate, ACM—NAM, ACM–NAM-hydrate and the piperazine salt of ACM exhibit the type I conformation, whereas ACM polymorphs and other cocrystals adopt the ACM type II conformation. Hydrogen-bond interactions in all the crystal structures were quantified by calculating their molecular electrostatic potential (MEP) surfaces. Hirshfeld surface analysis of the cocrystal surfaces shows that about 50% of the contribution is due to a combination of strong and weak O⋯H, N⋯H, Cl⋯H and C⋯H interactions. The physicochemical properties of these cocrystals are under study.

## Abstract 2

Acemetacin cocrystals were prepared by melt crystallization and their crystal structures determined by high-resolution powder X-ray diffraction. The difficult to obtain single crystals for the acemetacin family is overcome by structure determination from powder data.

## 1. Introduction   

Cocrystallization is a standard strategy to tailor physicochemical properties of drugs based on their chemical constituents (Childs *et al.*, 2004 ▸; Duggirala *et al.*, 2016 ▸; Bolla & Nangia, 2016 ▸) and supramolecular structure through crystal engineering (Desiraju *et al.*, 2011 ▸; Desiraju, 2013 ▸). Pharmaceutical cocrystals (Almarsson & Zaworotko, 2004 ▸; Schultheiss & Newman, 2009 ▸; Thakuria *et al.*, 2013 ▸) belong to a subclass of multicomponent systems in which one of the molecules must be an Active Pharmaceutical Ingredient (API) and the coformer is a Generally Regarded as Safe (GRAS) substance (http://www.fda.gov/Food/IngredientsPackaging­Labeling/GRAS/; accessed on 20/08/2016). Cocrystals incorporate pharmaceutically acceptable coformers and the drug substance into the same crystal lattice to provide a new composition of the API (Aitipamula *et al.*, 2012 ▸). Numerous cocrystal systems have been reported previously in more than a decade to modify the physicochemical and pharmacokinetic properties of drugs, notably solubility and bioavailability. The unique advantage of cocrystals is that they are amenable to those drugs which lack an ionizable functional group and thus present an alternative to the traditional salts for improving solubility and dissolution rate (Childs *et al.*, 2004 ▸; Bolla *et al.*, 2013 ▸), physical stability (Babu *et al.*, 2012 ▸; Trask *et al.*, 2006 ▸), bioavailability (Weyna *et al.*, 2012 ▸; Ganesh *et al.*, 2015 ▸), permeability (Sanphui, Devi *et al.*, 2015 ▸) and mechanical properties (Sun & Hou, 2008 ▸; Sanphui, Mishra *et al.*, 2015 ▸). However, certain drugs can be difficult to crystallize as single crystals, and one such example in our experience is acemetacin, whether it is the pure drug or its cocrystals. Structure solution from powder diffraction data for acemetacin cocrystals is reported in this paper as part of our continuing studies on this system (Sanphui *et al.*, 2013 ▸, 2014 ▸).

Acemetacin (ACM) is a glycolic acid ester prodrug of indomethacin and belongs to the non-steroidal anti-inflammatory drug (NSAID) class. It is metabolized to indomethacin, which then acts as an inhibitor of cyclooxygenase to produce the anti-inflammatory effects. ACM is sold under the trade name Emflex as 60 mg capsules (Merck KGaA). Solid-state forms of acemetacin have been studied by Chávez-Piña *et al.* (2007 ▸), Yoneda *et al.* (1981 ▸), Burger & Lettenbichler (1993 ▸) and Gelbrich *et al.* (2007 ▸). In our previous findings (Sanphui *et al.*, 2013 ▸, 2014 ▸), the crystal structures of ACM Form I and II were identified as synthon polymorphs of carboxylic acid dimer and catemer motifs. The binary adducts of cocrystals with nicotinamide (NAM), isonicotinamide (INA), picolinamide (PAM) and caprolactam (CPR) are stabilized by acid–amide hetero synthons and the *p*-aminobenzoic acid (PABA) cocrystal has the hetero acid dimer synthon. We noted that ACM tends to form a hydrate during any kind of solution-based cocrystal preparation, and so its crystallization was carried out in strictly anhydrous melt conditions (solventless). The structures of ACM Form I, ACM–INA and ACM–PABA were solved using single-crystal X-ray diffraction and those of Form II polymorph and cocrystals ACM–PAM, ACM–CPR, salt ACM–PPZ were solved by high-resolution powder X-ray diffraction data [Scheme 1, where superscript *a* indicates coformers reported in a previous study (Sanphui *et al.*, 2014 ▸) and superscript *b* coformers reported in this study].
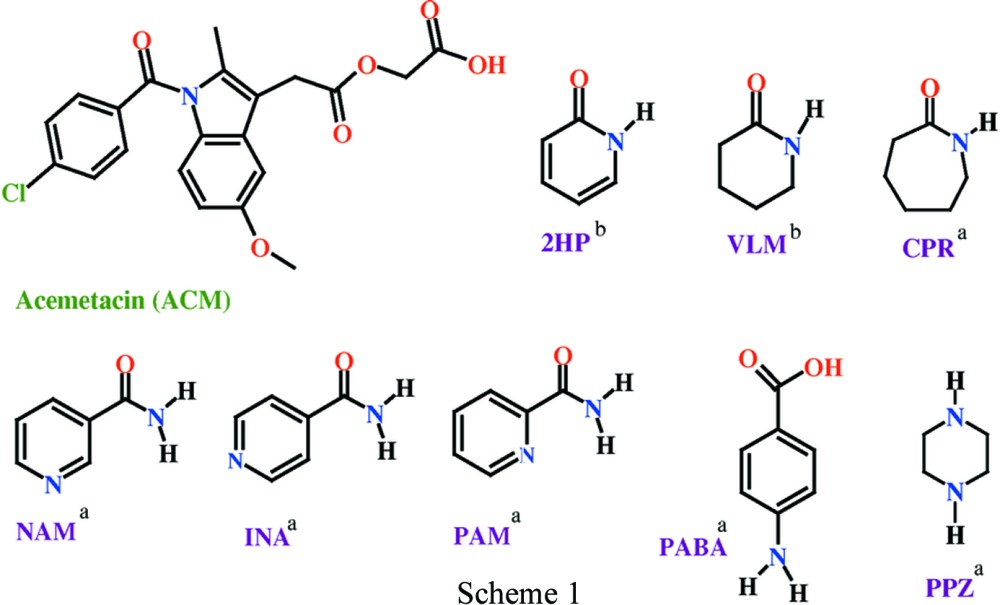


Among the binary systems reported in our previous papers, the ACM–NAM cocrystal was characterized by IR, powder X-ray diffraction (PXRD) and differential scanning calorimetry (DSC) but crystallization to obtain good diffraction quality single crystals was unsuccessful (Sanphui *et al.*, 2014 ▸), and structure determination from powder data (SDPD) was also not successful at that time. The cocrystal of ACM with *p*-aminobenzoic acid showed poor quality diffraction data and there was proton disorder in the ACM-COOH group. Therefore, high-resolution powder diffraction data were collected to solve the crystal structure of ACM–PABA and nicotinamide cocrystals.

Single-crystal X-ray diffraction is undoubtedly the most powerful tool to elucidate the molecular structure. However, the requirement for single crystals of appropriate size and quality limits the scope of this technique, because many materials crystallize as microcrystalline powders. Fortunately, there have been rapid advances during the past two decades in structure determination from powder diffraction data (SDPD; Harris *et al.*, 1994 ▸; Chernyshev, 2001 ▸; Harris, 2003 ▸; Le Bail *et al.,* 2009 ▸). SDPD is the method of choice when there is difficulty obtaining the optimum size single crystals and also when phase transformation, hydration or solvate formation issues complicate the isolation of good quality single crystals for data collection. Recently Ueto *et al.* (2012 ▸) reported furosemide−nicotinamide cocrystal polymorphs and cocrystal hydrate crystal structures solved from high-resolution powder data. The crystal structures of several API forms with three-dimensional coordinates determined have been reported using high-resolution powder data (David & Shankland, 2008 ▸; Braga *et al.*, 2012 ▸; Chernyshev *et al.*, 2013 ▸). In this background, we report crystal structures of acemetacin cocrystals listed in Scheme 1 (part b) from high-resolution powder diffraction data.

## 2. Experimental   

### 2.1. Preparation of acemetacin cocrystals/salts   

Acemetacin was purchased from Dalian Hong Ri Dong Sheng Import & Export Co. Ltd, China, http://dlhongridongsheng.guidechem.com/ and used as such without further purification. All the coformers were purchased form Sigma-Aldrich, India, and solvents are of analytically pure grade. ACM and the appropriate coformer was weighed in a 1:1 stoichiometric ratio in a 25 ml beaker and melted at 160°C. Cooling of the melt gave a glassy phase at room temperature (30°C) after 1–2 h, which was crystallized from different solvents, *e.g.* methyl isobutyl ketone (MIBK) and EtOAc. The solvents must be anhydrous (dry) to avoid the formation of ACM hydrates as by-products in crystallization. ACM–NAM-I, ACM–NAM-H, ACM–VLM, ACM–2HP and ACM–PABA cocrystals were prepared by melt crystallization. ACM–PABA was crystallized from dry EtOAc solvent. The purity and homogeneity of phases was confirmed by DSC (single endotherm).

### 2.2. Powder X-ray diffraction   

Bulk samples were analyzed by powder X-ray diffraction using a Bruker AXS D8 powder diffractometer (Bruker-AXS, Karlsruhe, Germany). Experimental conditions: Cu *K*α radiation (λ = 1.5418 Å); 40 kV, 30 mA; scan range 5–50° 2θ. High-resolution X-ray powder diffraction data for ACM–NAM-I, ACM–NAM-H, ACM–2HP, ACM–PABA and ACM–VLM were collected at room temperature using a Huber G670 Guinier camera with an image plate detector and Cu *K*α_1_ radiation (λ = 1.5406 Å). The unit-cell dimensions were determined using three indexing programs: *TREOR*90, *ITO* and *AUTOX* (Werner *et al.*, 1985 ▸; Visser, 1969 ▸; Zlokazov, 1992 ▸, 1995 ▸). The crystal structures were solved using the simulated annealing technique (Zhukov *et al.*, 2001 ▸) and refined using *MRIA* (Zlokazov & Chernyshev, 1992 ▸) following the procedure published earlier (Sanphui *et al.*, 2014 ▸). The initial molecular models for acemetacin and coformer molecules were taken from the Cambridge Structural Database (*ConQuest*, Version 1.18 with updates; Groom & Allen, 2014 ▸). In simulated annealing runs (without H atoms), the total number of degrees of freedom was either 20 or 21, *i.e.* 14 parameters for the acemetacin molecule (three translational, three rotational and eight torsional) and six or seven parameters for the coformer were varied. For ACM–NAM-H, the presence of solvent in the asymmetric part was approximated by a water molecule disordered over two positions. The occupancies were fixed to 0.5 s.o.f. and powder data collection, refinement parameters, hydrogen-bonding values are given in Tables 1 ▸ and 2 ▸. The diffraction profiles after the final bond-restrained Rietveld refinement are shown later in the paper. *X-Seed* (Barbour, 2001 ▸) was used to prepare the figures and packing diagrams.

### 2.3. Thermal analysis   

Differential Scanning Calorimetry (DSC) was performed on a Mettler Toledo DSC 822e module. Samples were placed in crimped but vented aluminium sample pans, with a typical sample size of 2–5 mg. The temperature range was 30–200°C at a heating rate of 5°C min^−1^. Samples were purged with a stream of dry N_2_ flow at 80 ml min^−1^.

### 2.4. Solid-state NMR spectroscopy   

Approximately 100 mg of fine crystalline sample was tightly packed into a zirconia rotor with the help of Teflon stick up to the cap Kel-F mark. A cross-polarization, magic angle spinning (CP-MAS) pulse sequence was used for spectral acquisition. Each sample was spun at a frequency of 5.0 ± 0.01 kHz and the magic angle setting was calibrated by the KBr method. Each data set was subjected to a 5.0 Hz line-broadening factor and subsequently Fourier transformed and phase corrected to produce a frequency domain spectrum. Solid-state ^13^C NMR spectra were obtained on a Bruker (Bruker BioSpin, Karlsruhe, Germany) Ultrashield 400 spectrometer utilizing a ^13^C resonant frequency of 100 MHz (magnetic field strength of 9.39 T). The chemical shifts were referenced to trimethylsilyl (TMS) using glycine (δ_glycine_ = 43.3 p.p.m.) as an external secondary standard. ^15^N CP-MAS spectra recorded at 400 MHz were referenced to glycine N and then the chemical shifts were recalculated to nitromethane (δ_glycine_ = −347.6 p.p.m.).

## 3. Results and discussion   

**Figure 1:**
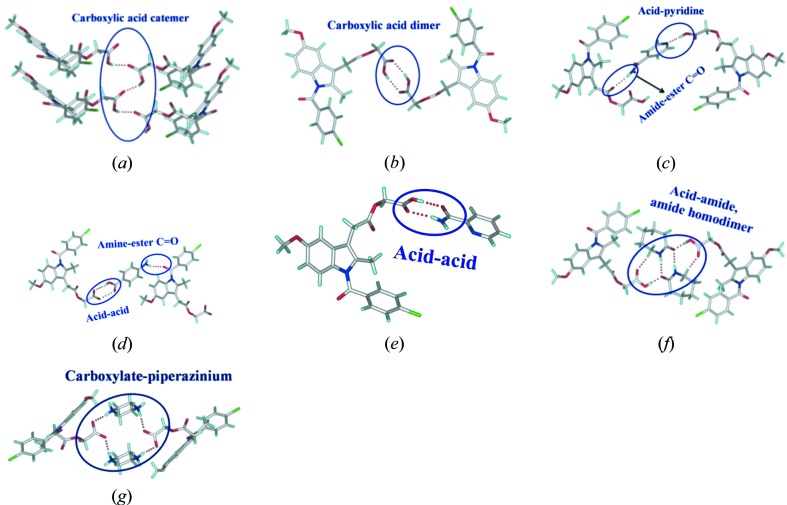
(*a*) O—H⋯O carboxylic acid catemer chain in ACM Form I. (*b*) Carboxylic acid dimer in ACM Form II. (*c*) to (*g*) The primary supramolecular synthons present in binary cocrystals ACM–INA, ACM–PABA, ACM–PAM, ACM–CPR and ACM–PPZ (Sanphui *et al.*, 2014 ▸).

The chemical units present in the crystal structures of Form I, II and cocrystals with INA, PAM, PABA are displayed in Fig. 1 ▸. Experimental conditions to yield single crystals in different solvents always resulted in either ACM hydrate or a precipitate. We therefore used the microcrystalline sample to solve the crystal structures from powder X-ray data collected at high resolution. The crystal structures of ACM–NAM were determined as two forms, an anhydrate ACM–NAM-I and a cocrystal hydrate ACM–NAM-H. X-ray crystal structure parameters are summarized in Table 1 ▸ and hydrogen-bond metrics in Table 2 ▸. The binary adducts were prepared by melt crystallization and characterized by their melting point, PXRD and DSC. The bulk phase purity was checked by PXRD Rietveld refinement and DSC.

### 3.1. Crystal structures of binary cocrystals   

#### 3.1.1. ACM–NAM-I (1:1)   

**Figure 2:**
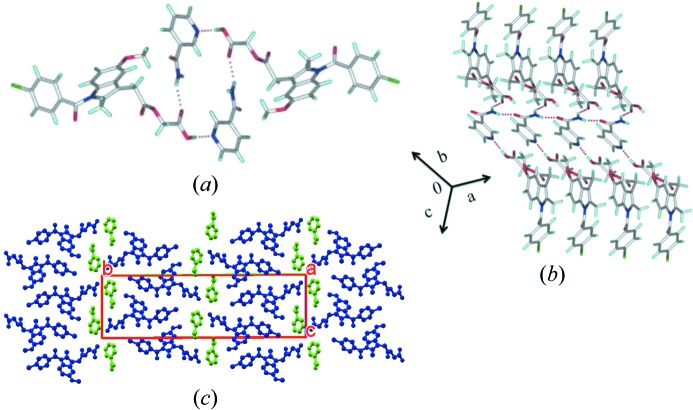
ACM–NAM-I. (*a*) Acid–pyridine and amide–acid synthons. (*b*) NAM coformers extend through amide chains and also interact with ACM to result in a sandwich-type packing. (*c*) NAM and ACM domains along the 2_1_ screw axis. H atoms are removed for clarity.

ACM–NAM-I (1:1) crystallized in the monoclinic crystal system *P*2_1_/*c*. The molecular packing is stabilized by an acid–pyridine primary synthon and further by an amide–acid hydrogen bond (Fig. 2 ▸ *a*). NAM molecules are bonded through amide catemer chains along the 2_1_ screw axis and also interact with ACM molecules to give a sandwich-type packing (Fig. 2 ▸ *b*). The two-dimensional packing of the asymmetric unit in Fig. 2 ▸(*c*) shows the separation of ACM and NAM domains in the structure.

#### 3.1.2. ACM−NAM-H (1:1:1)   

**Figure 3:**
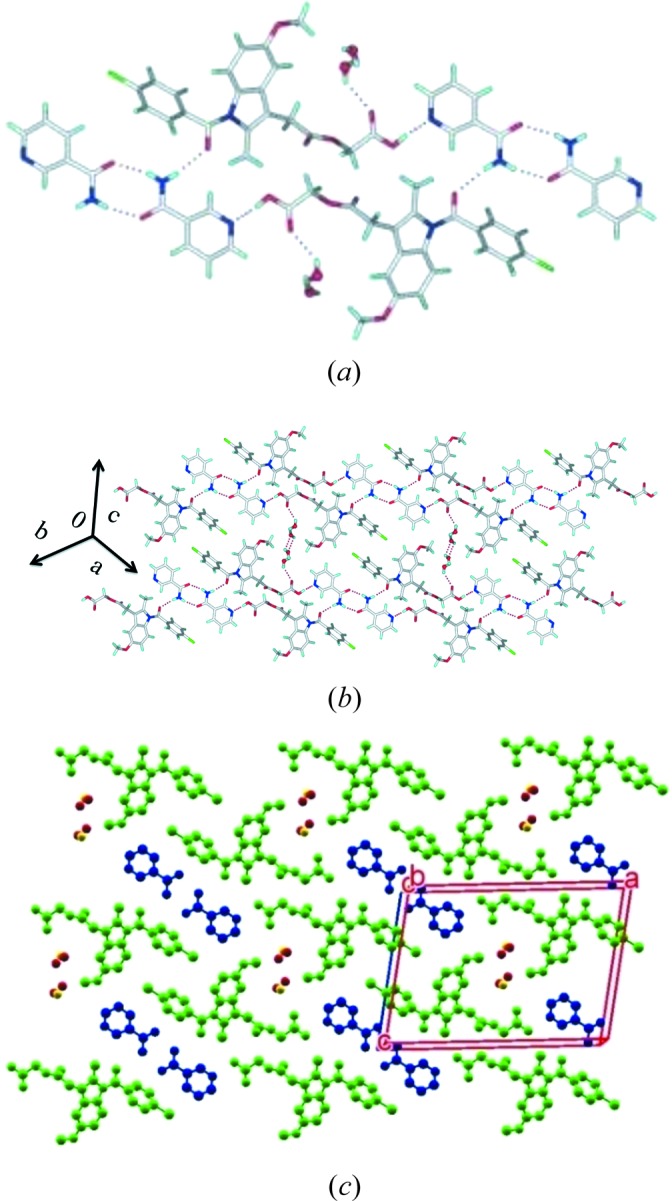
ACM–NAM-H (1:1:1) is a cocrystal hydrate. (*a*) Robust acid–pyridine synthon and NAM amide homodimers. (*b*) Two hydrogen-bonded layers extended through a water molecule in the crystal lattice. (*c*) The water molecule stoichiometry and disorder in crystal structure are confirmed by SDPD. H atoms are removed for clarity.

ACM–NAM-H is a hydrated form of ACM–NAM, wherein ACM, NAM and H_2_O cocrystallize in an equimolar ratio in the crystal structure of the space group 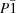. The acid–pyridine synthon, similar to that observed in ACM–NAM, is observed (Fig. 3 ▸ *a*) and furthermore NAM amide homodimers are present here, in contrast to the amide catemer chain in the anhydrate. The *anti*-N—H of NAM forms N—H⋯O hydrogen bonds with the amide group of ACM (Fig. 3 ▸ *b*). Water molecules are present in the (001) plane in a disorder state split over two sites with s.o.f. of 0.6 and 0.4, which extend *via* the O—H⋯O=C H bond with the acid group of ACM. The water molecules act as spacers between different layers along the *c*-axis (Fig. 3 ▸ *c*).

#### 3.1.3. ACM–VLM (1:1)   

**Figure 4:**
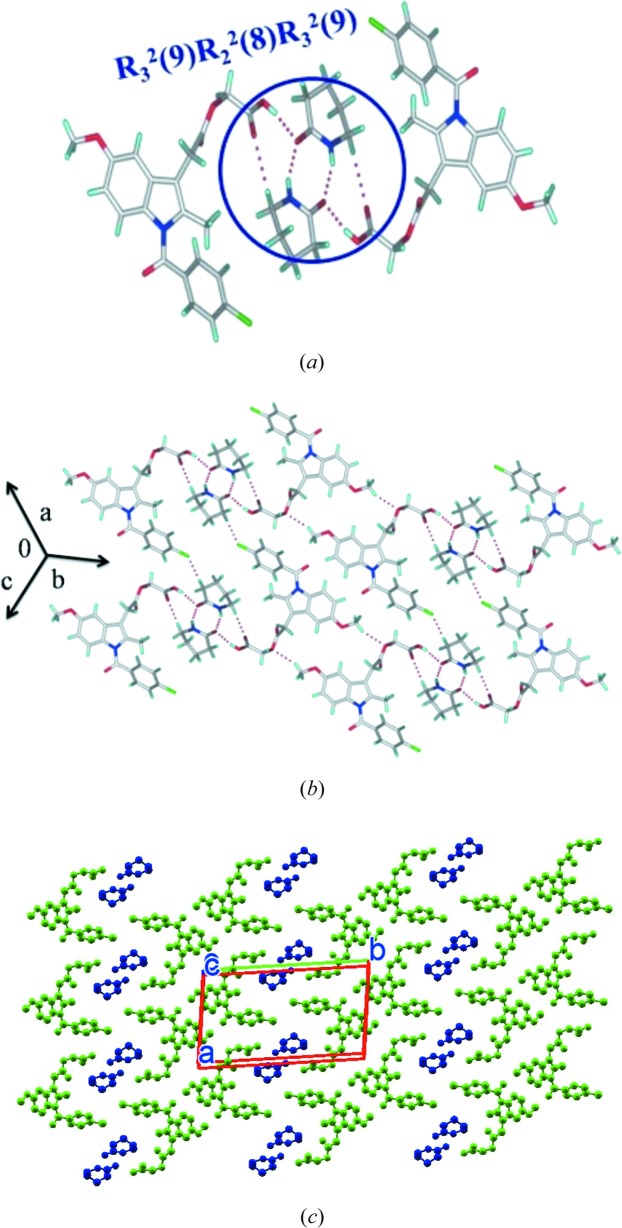
(*a*) Amide–amide homosynthon of VLM along with O—H⋯O H bonds in ACM–VLM results in an acid–amide three point synthon. (*b*) This synthon extends through weak C—H⋯Cl interactions. (*c*) Two-dimensional packing is displayed without H atoms for clarity.

The ACM–VLM (1:1) cocrystal in the 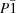 space group consists of an acid–amide three-point synthon (Fig. 4 ▸ *a*) *R*_3_^2^(9)*R*_2_^2^(8)*R*_3_^2^(9) graph-set motif (Etter *et al.*, 1990 ▸; Bernstein *et al.*, 1995 ▸) with VLM and is similar to the caprolactam cocrystal from our previous report (Sanphui *et al.*, 2014 ▸) (see Fig. 1 ▸ *f*). The *R*_3_^2^(9)*R*_2_^2^(8)*R*_3_^2^(9) motif extends in the (010) plane with C—H⋯O and C—H⋯Cl interactions (Fig. 4 ▸ *b*). VLM molecules form sandwiches with ACM molecules in the crystal structure along the *c*-axis (Fig. 4 ▸ *c*).

#### 3.1.4. ACM–2HP (1:1)   

**Figure 5:**
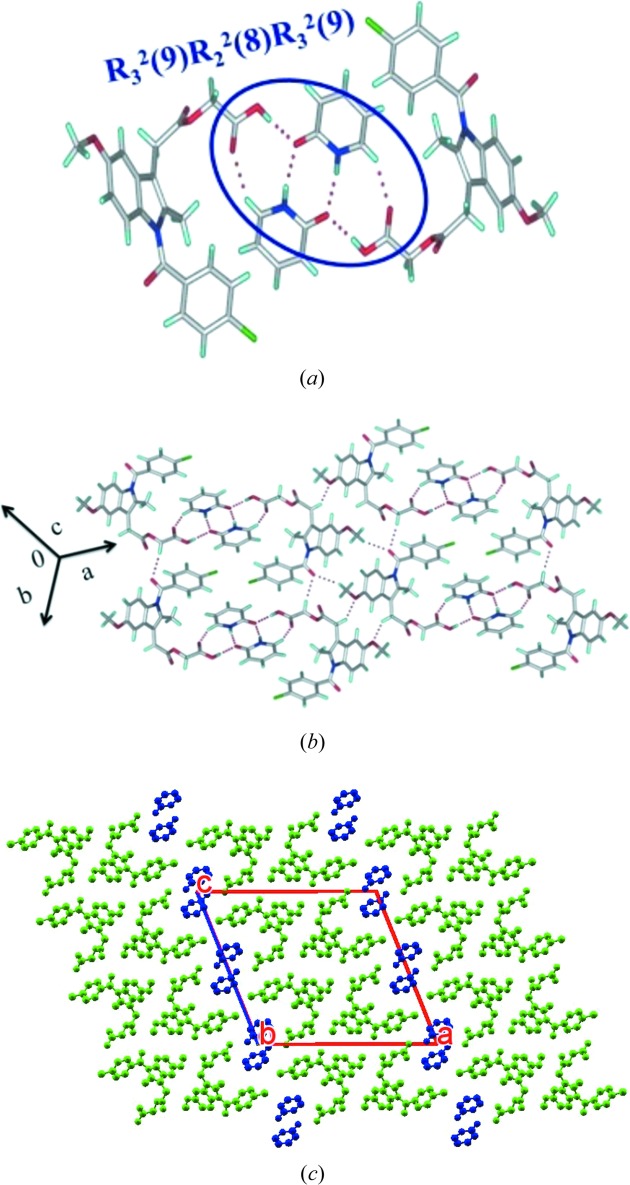
ACM–2HP (1:1). (*a*) Amide–amide homosynthon of 2HP along with O—H⋯O hydrogen bonds with ACM to give the acid–amide three-point synthon. (*b*) The extended hydrogen-bond network in the crystal structure. (*c*) Two-dimensional sandwich packing. H atoms are removed for clarity.

ACM–2HP (1:1) crystallized in the *P*2_1_/*c* space group *via* the acid–amide three-point synthon *R*_3_^2^(9)*R*_2_^2^(8)*R*_3_^2^(9) (Fig. 5 ▸ *a*), similar to that in CPR and VLM cocrystals. These synthons extend *via* C—H⋯O interactions with adjacent ACM molecules *via* glycolate ester CH_2_ and amide C=O to result in a layered packing (Fig. 5 ▸ *b*). 2HP molecules are sandwiched with ACM molecules along the *c*-axis (Fig. 5 ▸ *c*).

#### 3.1.5. ACM–PABA (1:1)   

**Figure 6:**
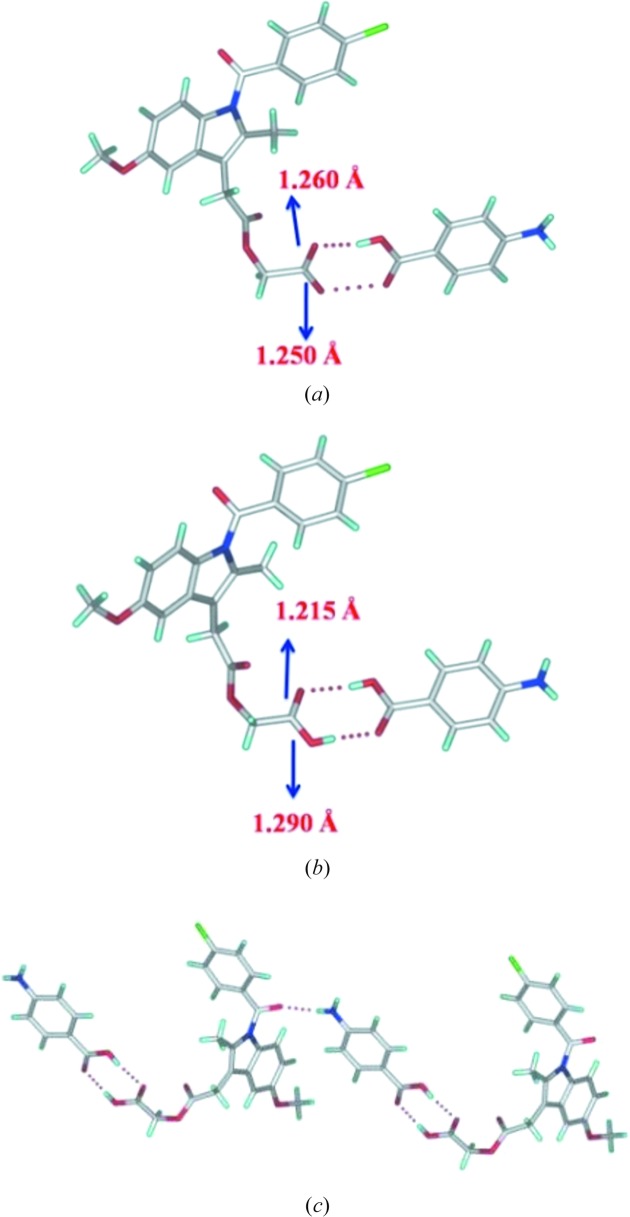
(*a*) ACM–PABA. (*a*) Previously reported structure (Sanphui *et al.*, 2014 ▸). (*b*) SDPD crystal structure with better precision C=O and C—O distance for the COOH group (this paper). (*c*) Bond lengths of the ACM carboxylic acid group mean that the heterodimer of COOH groups and N—H⋯O H bonds are present in ACM–PABA. The unit-cell parameters are similar indicating no polymorphism.

The ACM–PABA (1:1) crystal structure has been reported by us previously (Sanphui *et al.*, 2014 ▸). However, the diffraction quality of the tiny needle-shape single crystals was not good enough and so proton disorder in the COOH group and C=O, C—O distances could not be measured to a high enough precision (Fig. 6 ▸ *a*). In order to resolve this issue, we revisited the ACM–PABA structure by SDPD. The bond distances of the COOH group in ACM and PABA are now measured accurately and show that the COOH group is present as a neutral group to confirm that the structure is a cocrystal (and not a salt or salt-cocrystal; Fig. 6 ▸ *b* and *c*). The significance of the SDPD technique is demonstrated in this cocrystal structure.

All crystallographic parameters and hydrogen bond distances are listed in Tables 1 ▸ and 2 ▸.

### 3.2. Conformational analysis   

**Figure 7:**
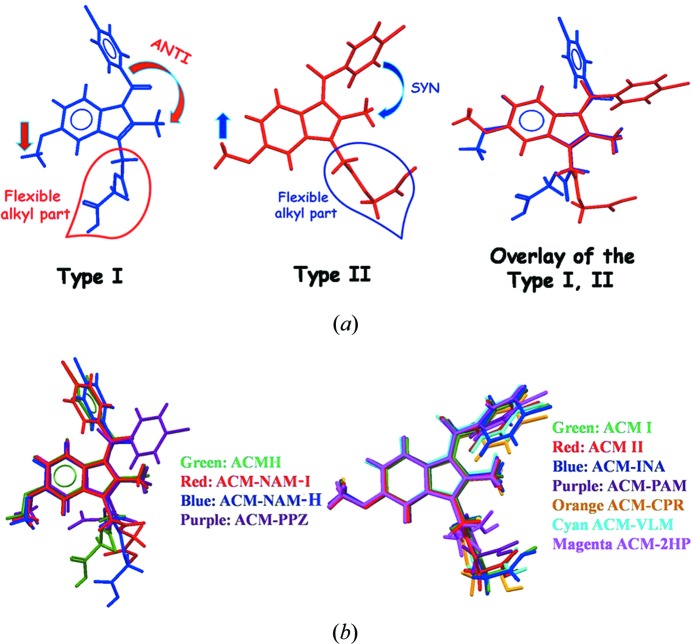
(*a*) Classification of the conformations present in ACM as Type I and II. Molecular overlay of ACMH Type I conformation in NAM, PPZ binary cocrystals (left) and ACM form I in Type II conformation (right) and the binary adducts indicates torsional flexibility of the carboxamide and alkyl chain in the glycolic acid ester. (*b*) The left side is the overlay of ACM Form I and cocrystals in the present study and the right side is the results from a previous study (Sanphui *et al.*, 2014 ▸).

The alkyl chain, glycolic ester, *p*-Cl-benzoyl group and OMe groups attached to the planar indole ring exhibit conformational flexibility. The rotations about C—C bonds (Fig. 7 ▸ *a*) are classified as Type I or II. The orientation of the *p*-Cl-benzoyl and OMe group in ACM hydrate (Fig. 7 ▸ *b*) match with that of ACM–NAM-I, ACM–NAM-H in Type I conformation, whereas the other cocrystals match with Form I ACM labeled as Type II. The orientation of the OMe group of ACM–PPZ adopts a parallel conformation with ACMH (Type I) and the *p*-Cl-benzoyl group exhibits good similarity with ACM Form I (Type II), and it resides in the middle of Type I and II. The alkyl chain part such as glycolic acid is flexible (Fig. S2 of the supporting information) and shows variable conformations in the structures (torsion angles are listed in Table S2). ACMH, ACM–NAM-I and ACM–NAM-H adopt the same conformation (Type I), whereas the cocrystals ACM–PABA, ACM–PAM, ACM–INA, ACM–CPR, ACM–VLM and ACM–2HP are in parallel conformation with ACM Form I (Type II); the PPZ salt is in between the two conformations. The strong hydrogen-bonding synthons result in conformation changes to guide the overall packing, but a detailed understanding of conformation changes with packing forces (intra- and intermolecular) in crystal structures is still elusive.

### 3.3. PXRD and DSC analysis of binary cocrystals   

**Figure 8:**
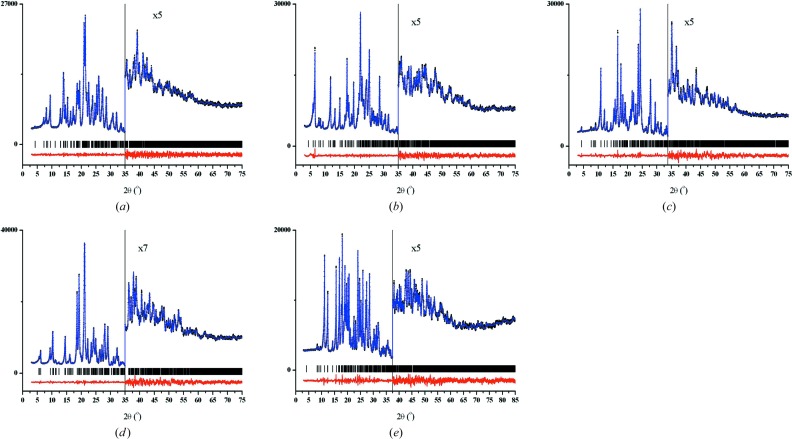
PXRD plots by Rietveld refinement. (*a*) ACM–NAM-I, (*b*) ACM–NAM-H, (*c*) ACM–VLM, (*d*) ACM–2HP and (*e*) ACM–PABA showing the experimental (black dots), calculated (blue) and difference (red) curves of powder XRD. The vertical bars denote calculated positions of the diffraction peaks.

The products of cocrystallization were characterized by their powder XRD pattern and the overlay of experimental line profile on the calculated lines from the crystal structure (Fig. 8 ▸). Apart from ACM–NAM which is polymorphic, all other cocrystals were crystallized in a single phase.

**Figure 9:**
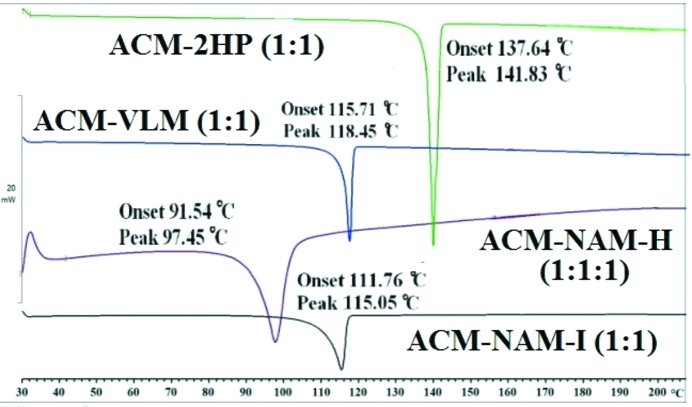
ACM cocrystals exhibit a single endotherm in DSC.

Crystallization of ACM–NAM melted solid from solvents such as methyl isobutyl ketone and methyl ethyl ketone gave Form I, whereas dry EtOAC, acetonitrile, resulted in a hydrate (ACM–NAM-H). PXRD of ACM–NAM-I and ACM–NAM-H are different. A broad endotherm was observed at 90–100°C for ACM–NAM-H, whereas Form I starts to melt at 111°C (Fig. 9 ▸). Since DSC shows melting below 100°C and a single endotherm, our preliminary assumption was these two products are polymorphs. After solving the crystal structure from SDPD the same result was confirmed in that Form I is anhydrate (ACM–NAM-I), whereas Form II is a hydrate (ACM–NAM-H). The existence of the water in crystal lattice was proven by SDPD to show that water loss from the hydrate and melting occurs simultaneously in this compound. ACM–VLM and ACM–2HP were similarly characterized by DSC in the bulk phase (Fig. 9 ▸).

### 3.4. Solid-state NMR spectroscopy   

**Figure 10:**
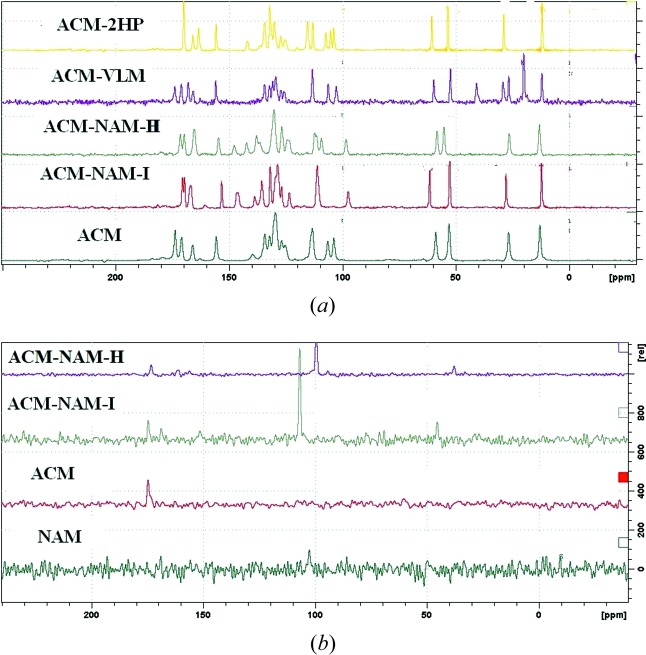
(*a*) ^13^C ss-NMR spectra of acemetacin cocrystals. Small differences were observed in the chemical shifts of peaks compared with the starting compounds. (*b*) ^15^N ss-NMR spectra of acemetacin cocrystals with ACM-NAM Form I and hydrate exhibiting significant differences in their ^15^N NMR spectra.

Solid-state NMR (Tishmack *et al.*, 2003 ▸; Widdifield *et al.*, 2013 ▸) is an informative tool to characterize cocrystals. The purpose of the NMR experiments was twofold: to confirm the molecular structure of the cocrystal and its stoichiometry, and to confirm the proton state in terms of salt-cocrystal state. Such questions are best answered by ^15^N NMR spectroscopy because the chemical shift of neutral and ionic NH^+^ will be very different. Three distinct carbonyl peaks exist for ACM (carboxylic acid, ester and carboxamide). The coformers NAM, VLM and 2HP have a C=O bond group also, which makes it extremely challenging to assign carbon peaks unambiguously in ^13^C ss-NMR spectra (Fig. 10 ▸ *a*; δ values are listed in Table S1). The presence of four different carbonyl peaks in the 150–180 p.p.m. region is characteristic of ACM–VLM, whereas ACM–NAM-I and the hydrate exhibit a difference of 51–62 p.p.m. in the aromatic region. ^15^N ss-NMR spectra were recorded, but the peak intensities were extremely low. There is a clear shift observed in ^15^N ss-NMR, *e.g.* NAM peak at 102.5 shifted to 106.8 in Form I and 99.4 p.p.m. in the hydrate form (Fig. 10 ▸ *b*, Table S1).

### 3.5. Hirshfeld surface analysis   

**Figure 11:**
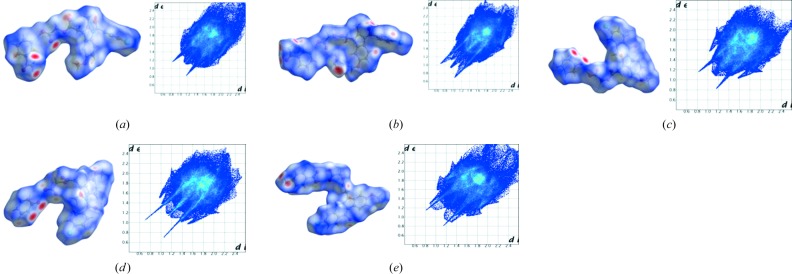
Hirshfeld surface analysis of ACM cocrystals along with their surface map and two-dimensional fingerplots. ^a^Cocrystals reported in this study, ^b^cocrystals report in previous study (Sanphui *et al.*, 2014 ▸).

**Figure 12:**
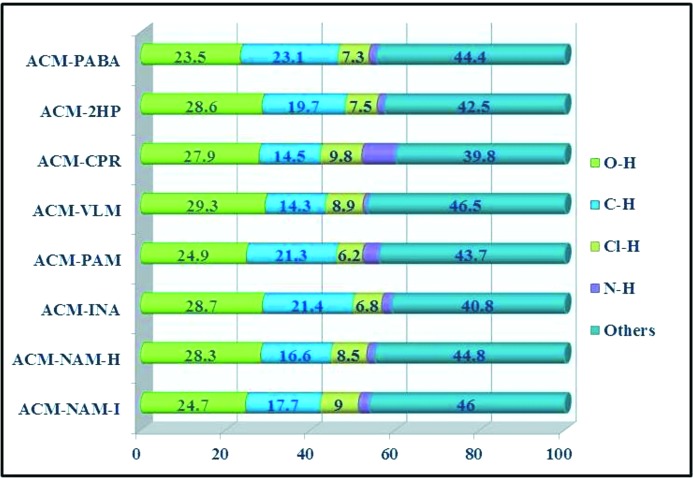
Percentage of intermolecular interactions in ACM cocrystals from Hirshfeld surface analysis.

Hirshfeld surface analysis (Hirshfeld, 1977 ▸; Spackman & Jayatilaka, 2009 ▸; Spackman & McKinnon, 2002 ▸) is related to the proximity of near neighbor molecules and the intermolecular interactions. Hirshfeld analysis allows a pictorial identification of the characteristic interactions throughout the structure. The fingerprint plots and surface analysis of ACM cocrystals are displayed in Fig. 11 ▸ and Fig. S1. Each crystal structure exhibits a unique fingerprint plot of weak interactions present in that particular system, and it is easy to differentiate the percentage of H⋯*X* hydrogen bond to hetero atom interactions. The Hirshfeld surface analysis shows that O⋯H, C⋯H, N⋯H and Cl⋯H interactions vary from one cocrystal structure to another (Fig. 12 ▸), and that their total contribution is less than 50%. The isotropic van der Waals and C—H⋯π, H⋯H, π⋯π interaction wings appear at the top of the fingerprint region. Among all the hetero interactions observed in this study, H⋯O has a major contribution to the two-dimensional fingerplots (Fig. 12 ▸).

## 4. Conclusions   

The advantage and ease of SDPD is successfully demonstrated in this study on acemetacin cocrystals. Cocrystals of ACM–PABA, ACM–NAM-I, ACM–NAM-H, ACM−VLM and ACM−2HP were prepared by melt crystallization and their crystal structures solved using three-dimensional parameters obtained from high-resolution powder X-ray data. ss-NMR spectroscopy enabled the identification of cocrystals and different forms of NAM based on the shift in ^13^C and ^15^N resonance values. The novel binary phases of ACM–NAM were prepared by solidification of the melt phase followed by recrystallization from anhydrous solvents in dry conditions. The observed proton disorder in PABA cocrystal, which was previous solved as a less accurate crystal structure, is now improved using high-resolution SDPD data. ACM–NAM-I, ACM–NAM-H are confirmed as anhydrate and hydrate forms by high-resolution powder data. DSC suggests single endotherms for both the forms and crystallization experiments for single crystals resulting in ACM hydrate, showing that SDPD is the method of choice to confirm the two forms. Hirshfeld surface analysis exhibits unique fingerplots for different solid phases and differences in wings and spikes for the novel phases. The contribution of OH interactions in these crystal structures is visually depicted in Hirshfeld plots.

## Supplementary Material

Crystal structure: contains datablock(s) ACM2HP, ACMNAMH, ACMNAMI, ACMPABA, ACMVLM. DOI: 10.1107/S2052252517002305/ed5011sup1.cif

Rietveld powder data: contains datablock(s) ACM2HP. DOI: 10.1107/S2052252517002305/ed5011ACM2HPsup2.rtv

Rietveld powder data: contains datablock(s) ACMNAMH. DOI: 10.1107/S2052252517002305/ed5011ACMNAMHsup3.rtv

Rietveld powder data: contains datablock(s) ACMNAMI. DOI: 10.1107/S2052252517002305/ed5011ACMNAMIsup4.rtv

Rietveld powder data: contains datablock(s) ACMPABA. DOI: 10.1107/S2052252517002305/ed5011ACMPABAsup5.rtv

Rietveld powder data: contains datablock(s) ACMVLM. DOI: 10.1107/S2052252517002305/ed5011ACMVLMsup6.rtv

Supporting tables and figures. DOI: 10.1107/S2052252517002305/ed5011sup7.pdf

CCDC references: 1507491, 1507492, 1507493, 1507494, 1507495

## Figures and Tables

**Table 1:** Crystallographic details of ACM cocrystals

	ACM–NAM-I	ACM–NAM-H	ACM–VLM	ACM–2HP	ACM–PABA
CCDC No.	1507493	1507492	1507495	1507491	1507494
Chemical formula	C_21_H_18_ClNO_6_·C_6_H_6_N_2_O	C_21_H_18_ClNO_6_·C_6_H_6_N_2_O·H_2_O	C_21_H_18_ClNO_6_·C_5_H_9_NO	C_21_H_18_ClNO_6_·C_5_H_5_NO	C_21_H_18_ClNO_6_·C_7_H_7_NO_2_
*M*_r_	537.94	555.96	514.95	510.91	552.95
Crystal system	Monoclinic	Triclinic	Triclinic	Monoclinic	Monoclinic
Space group	*P*2_1_/*c*	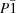	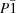	*P*2_1_/*c*	*P*2_1_
*a* (Å)	4.8977 (11)	21.348 (2)	11.764 (12)	23.1400 (18)	17.294 (18)
*b* (Å)	40.914 (4)	4.1931 (12)	20.555 (19)	5.1900 (8)	4.819 (7)
*c* (Å)	12.8874 (19)	15.2174 (19)	5.1627 (9)	21.2642 (19)	16.955 (15)
α (°)	90	90.567 (17)	89.543 (14)	90	90
β (°)	100.328 (18)	101.40 (2)	93.300 (16)	111.714 (17)	113.529 (17)
γ (°)	90	89.473 (16)	96.276 (17)	90	90
*V* (Å^3^)	2540.6 (7)	1335.2 (4)	1238.8 (3)	2372.5 (5)	1295.5 (3)
*M*_20_	36	24	21	21	21
*F*_30_	61 (0.008, 64)	47 (0.010, 44)	39 (0.010, 53)	39 (0.010, 53)	39 (0.010, 53)
*Z*	4	2	2	4	2
ρ_calc_ (g cm^−3^)	1.406	1.383	1.380	1.430	1.417
μ (mm^−1^)	1.784	1.743	1.787	1.866	1.783
2θ_min_–2θ_max_, increment (°)	3.00–75.00, 0.01	3.00–75.00, 0.01	3.00–75.00, 0.01	3.00–85.00, 0.01	3.00–75.00, 0.01
Number of parameters, restraints	195, 125	203/125	187/123	187/121	197/131
*R*_p_/*R*_wp_/*R*_exp_	0.0160/0.0180/0.0167	0.0161/0.0191/0.0160	0.0208/0.0271/0.0175	0.0205/0.0265/0.0185	0.0183/0.0236/0.0173
Goodness-of-fit	1.076	1.198	1.549	1.433	1.365

**Table 2:** Hydrogen-bond geometry (Å, °) in crystal structures

	*D*—H⋯*A*	H⋯*A*	*D*—H⋯*A*	*D*—H⋯*A*
ACM–NAM-I	O6–H6*A*⋯N3	1.71	2.532 (15)	175
	N2—H2*A*⋯O5^i^	2.13	2.866 (15)	144
	N2—H2*B*⋯O7^ii^	2.13	2.938 (15)	157
ACM–NAM-H	O6—H6*A*⋯N3	1.72	2.530 (14)	168
	N2—H2*A*⋯O7^iii^	2.05	2.881 (14)	163
	N2—H2*B*⋯O1^iv^	2.15	2.999 (14)	167
ACM–VLM	O6—H6*A*⋯O7	1.92	2.706 (12)	168
	N2—H2*A*⋯O7^vii^	2.12	2.921 (15)	156
ACM–2HP	O6—H6*A*⋯O7	1.74	2.550 (10)	168
	N2—H2*A*⋯O7^v^	1.90	2.739 (12)	166
ACM–PABA	O6—H6*A*⋯O8	1.79	2.581 (9)	161
	O7—H7⋯O5	2.00	2.811 (10)	172
	N2—H2*A*⋯O1^ii^	2.11	2.918 (15)	157
	N2—H2*B*⋯N2^vi^	2.33	3.168 (13)	166

Symmetry codes: (i) 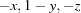; (ii) 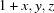; (iii) 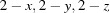; (iv) 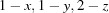; (v) 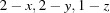; (vi) 
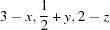
; (vii) 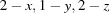.
