# Supplementary material for: Acemetacin cocrystal structures by powder X-ray diffraction
Source: IUCrJ. 2017 Mar 8;4(Pt 3):206–14. doi: 10.1107/S2052252517002305 (PMC5414395; doi:10.1107/S2052252517002305)
Supplement: Supplementary file 7 [file m-04-00206-sup7.pdf]

IUCrJ

Volume 4 (2017)

Supporting information for article:

Acemetacin cocrystal structures by powder X-ray diffraction

Geetha Bolla, Vladimir Chernyshev and Ashwini Nangia

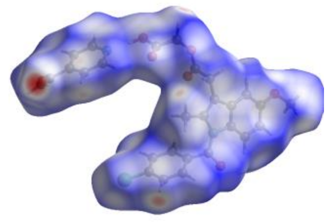	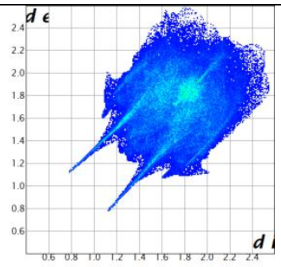	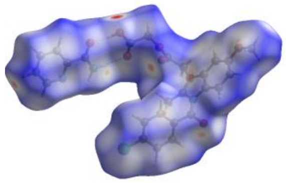	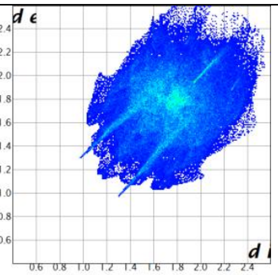
(a) ACM−INA[b]		(b) ACM−PAM[b]	
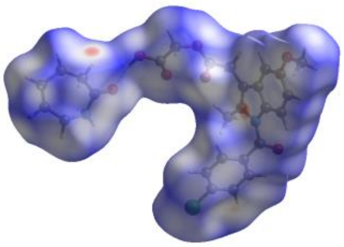		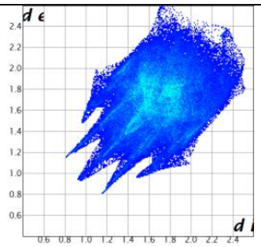	
(c) ACM−CPR[b]			

[a] cocrystals report in previous study (Sanphui *et al.*, 2014), [a] cocrystals report in present study

Figure S1 Hirshfeld surface analysis of the ACM cocrystals along with their surface map and 2D finger plots.

Table S1 ^{13}C and ^{15}N-NMR ss-NMR δ values (ppm).

^{13}C ss-NMR values				
ACM	ACM−NAM-I	ACM−NAM-H	ACM−VLM	ACM−2HP
12.8	12.06	13.01	11.94	11.82
26.56	27.71	26.32	19.88	28.71
52.83	52.58	55.04	26.53	53.37
58.68	61.40	58.10	29.10	60.45
103.60	97.35	98.31	40.66	103.61
106.32	110.98	109.96	52.31	105.08
113.16	123.32	111.37	59.57	107.21
125.08	126.72	112.16	102.56	113.03
126.73	128.54	124.13	106.14	115.28
129.49	131.76	126.60	113.18	120.00
132.06	135.39	130.06	125.68	125.19
134.18	145.96	136.18	127.01	127.12
139.40	153.08	137.76	129.34	129.94
158.48	160.18	142.15	130.72	131.99
165.99	160.72	147.59	132.09	134.27
170.78	166.74	154.51	134.32	141.79
173.64	169.44	165.28	155.72	155.61
	170.37	169.64	165.82	163.33
		171.34	167.93	165.87
			170.87	179.77
			173.77	

^{15}N ss-NMR values		
ACM	ACM−NAM-I	ACM−NAM-H
174.63	45.39	37.81
	106.86	99.44
	168.75	156.09
	174.59	173.21

Table S2 Torsion angles (°) variation in ACM crystal structures (see Fig. 8).

	τ_1	τ_2	τ_3	τ_4	τ_5
ACM Form I[a]	27.9	53.3	175.2	-179.9	78.4
ACM Form II[a]	35.7	38.0	-172.7	-172.3	-81.3
ACMH[a]	154.2	48.6	-7.8	-180.2	68.5
ACM-INA[a]	29.8	49.4	179.8	-178.0	79.9
ACM-PAM[a]	23.4	51.9	-179.9	-160.6	-96.4
ACM-CPR[a]	35.3	40.1	172.3	-160.1	-76.8
ACM-PABA[a]	43.6	40.6	179.5	-179.8	75.7
ACM-PPZ [a]	18.2	36.4	-46.9	-148.8	149.9
ACM-NAM -I[b]	-156.1	50.1	-78.2	-168.4	157.14
ACM-NAM-H[b]	-145.9	29.7	-170.86	168.7	-119.1
ACM-VLM[b]	-26.7	2.8	169.4	165.0	72.5
ACM-2HP[b]	19.5	56.9	-27.5	174.5	79.1
ACM-PABA[b]	-36.2	-46.6	171.1	175.8	-81.2

[a] cocrystals report in previous study (Sanphui *et al.*, 2014), [a] cocrystals report in present study

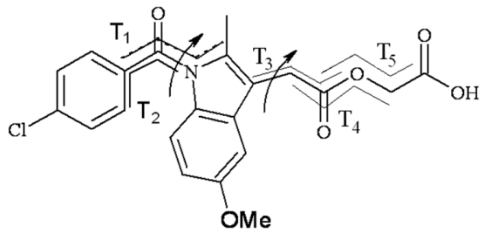

Figure S2 Flexible torsion angles in ACM (Sanphui *et al.*, 2014).

Reference

Sanphui, P., Bolla, G., Nangia, A., & Chernyshev, V. (2014). *IUCrJ* **1**, 136–150.
